# GmBICs Modulate Low Blue Light-Induced Stem Elongation in Soybean

**DOI:** 10.3389/fpls.2022.803122

**Published:** 2022-02-03

**Authors:** Ruolan Mu, Xiangguang Lyu, Ronghuan Ji, Jun Liu, Tao Zhao, Hongyu Li, Bin Liu

**Affiliations:** The National Key Facility for Crop Gene Resources and Genetic Improvement (NFCRI), Institute of Crop Science, Chinese Academy of Agricultural Sciences, Beijing, China

**Keywords:** shade avoidance syndrome, stem elongation, BIC, CRISPR/Cas9, soybean, low blue light

## Abstract

Blue-light inhibitors of cryptochromes (BICs) promote hypocotyl elongation by suppressing the activity of cryptochromes in *Arabidopsis*. Nevertheless, the roles of BICs in other plant species are still unclear. Here we investigate their functions by genetic overexpression and CRISPR/Cas9 engineered mutations targeting the six *GmBIC* genes in soybean. We showed that the *GmBICs* overexpression (*GmBICs-OX*) lines strongly promoted stem elongation, while the single, double, and quadruple mutations in the *GmBIC* genes resulted in incremental dwarfing phenotypes. Furthermore, overexpression of GmBIC2a abolished the low blue light (LBL)-induced stem elongation, demonstrating the involvement of GmBICs in regulating cryptochrome-mediated LBL-induced shade avoidance syndrome (SAS). The *Gmbic1a1b2a2b* quadruple mutant displayed reduced stem elongation under LBL conditions, which was reminiscent of the *GmCRY1b-OX* lines. Taken together, this study provided essential genetic resources for elucidating GmBICs functional mechanisms and breeding of shade-tolerant soybean cultivars in future.

## Introduction

*Arabidopsis thaliana* harbors two cryptochromes, CRY1 and CRY2, which mediate the blue-light-dependent inhibition of hypocotyl elongation and photoperiodic flowering, respectively (Ahmad and Cashmore, 1993; Guo et al., 1998; Sancar, 2000; Cashmore, 2003). To maintain cell photosensitivity, photoreceptors are usually deactivated by various negative feedback mechanisms post receiving light illumination. For example, the light-activated cryptochrome undergoes degradation through the ubiquitin-proteasome pathway in both plants and animals (Busino et al., 2007; Yu et al., 2007; Hirota et al., 2012; Xing et al., 2013).

The direct inhibiting factor of cryptochrome was first characterized by screening the *Arabidopsis* FOX (full-length cDNA overexpression gene hunting system) library (Ichikawa et al., 2006). Three FOX lines overexpressing the same gene were identified to be phenocopy with the *cry1cry2* mutant, including elongated hypocotyl under blue light, less anthocyanin accumulation, and delayed flower time under long-day conditions (Wang Q. et al., 2016). The casual gene and its homologous gene in *Arabidopsis* were named as *Blue-light Inhibitor of Cryptochromes 1* (*BIC1*, At3G52740) and *BIC2* (At3G44450), respectively. Both BIC1 and BIC2 are located in the cytosol and the nucleus containing a highly conserved Cryptochrome Interacting Domain (CID) among different species. Extensive studies showed that BIC interacts with the PHR domain of CRYs through the CID domain to inhibit the photoactivation or post-photoactivating processes of CRYs, including dimerization, phosphorylation, protein interaction, formation of photobodies, and degradation (Wang Q. et al., 2016; Ma et al., 2020; Wu et al., 2021).

The *CRY* and *BIC* genes form a negative feedback circuitry that regulates blue light sensitivity in *Arabidopsis* (Wang et al., 2017). The inactive CRY proteins present as monomers in dark conditions, while the photoactivated CRY proteins form active homodimers or oligomers, which interact with Suppressor of PhyA-105 (SPA), Phytochrome-Interacting Factor (PIF), and Cryptochrome-Interacting Basic Helix-loop-helixes (CIB) proteins to regulate downstream gene transcription, thus facilitating photomorphogenesis. Photoactivated CRY inhibits the Constitutive Photomorphogenic 1 (COP1)/SPA complex which destabilizes the transcriptional factor HY5 (Liu et al., 2011). The accumulated HY5 directly upregulates the expression of BIC which then prevents the dimerization of CRY. Thus, this negative feedback loop fine-tunes CRY activity to maintain cells with appropriate photosensitivity (Wang et al., 2017, 2018).

Soybean [*Glycine max* (L.) Merr.], as one of the economically important crops, displays obvious shade avoidance syndrome (SAS), especially the exaggerated stem elongation that results in lodging and yield reduction in response to the reduced blue light under density planting conditions (Pierik, 2021). Our previous studies showed that overexpression of cryptochrome could significantly improve the performance of soybean under shade conditions (Lyu et al., 2021). We surmise that the knockout of *GmBIC* genes may enhance the blue light signal transduction activity of GmCRYs, thus inhibiting the excessive stem elongation induced by low blue light. In this study, we obtained the GmBICs overexpression lines and CRISPR/Cas9 knockout mutants in soybean. Furthermore, we verified the function of *GmBIC* genes in regulating plant height and provided germplasms facilitating the breeding of shade-tolerant soybean cultivars.

## Materials and Methods

### Plant Materials and Growth Conditions

Soybean cultivar Tian Long 1 (TL1) was used as a wild-type control. The stable overexpression lines and CRISPR/Cas9 knockout lines were generated by the genetic transformation of TL1. For the tissue-specific expression analysis, the tissues including roots, stems, cotyledons, unifoliolate leaves, the first trifoliolate leaves, and apical tissues were taken from soybean cultivar Williams 82 (Ws82) grown in the plant growth chamber under continuous light for 14 days with three replicates for each sample. For the subcellular localization assay, the protoplasts were isolated from the leaves of Ws82 grown under the short-day conditions (8 h light/16 h dark) in the plant growth chamber with the light intensity of 120–180 μmol m^–2^ s^–1^ at the temperature of 25°C as described previously (Xiong et al., 2019). The homozygous overexpression lines and CRISPR/Cas9 knockout lines were selected and reproduced for at least four generations, and then used for phenotypic observation. For field testing, the indicated plants were grown in Beijing field with plant spacing 23.5 cm and row spacing 60 cm in 2020 and 2021, respectively.

### Primers and Accession Numbers

The primers involved in this study are listed in Supplementary Table 1. Gene sequences or protein sequences were obtained from the Phytozome database^1^ with corresponding accession numbers: *GmBIC1a* (*Glyma.10G072000*), *GmBIC1b* (*Glyma.13G153400*), *GmBIC1c* (*Glyma.19G194900*), *GmBIC1d* (*Glyma.03G195300*), *GmBIC2a* (*Glyma.12G184800*), *GmBIC2b* (*Glyma.13G316500*), *AtBIC1* (*AT3G52740*), *AtBIC2* (*AT3G44450*), *MtBIC1* (*Medt r2g086480*), *MtBIC2* (*Medtr7g104540*), *MtBIC3* (*Medtr1 g059990*), *SvBIC1* (*Sevir.7G098100*), *SvBIC2* (*Sevir.1G183600*), *OsBIC1* (*Os04g33610*), *OsBIC2* (*Os02g32990*), *SbBIC1* (*Sob ic.004G165500*), *SbBIC2* (*Sobic.006G068900*), *ZmBIC1* (*Zm00 001d025347*), *ZmBIC2* (*Zm00001d003799*), *ZmBIC3* (*Zm000 01d016698*), *GbBIC1* (*Gobar.D05G083700*), *GbBIC2* (*Gobar.A05 G085300*), *GbBIC3* (*Gobar.A01G219400*), *GbBIC4* (*Gobar.D01 G233800*), *GbBIC5* (*Gobar.D04G115400*), *GbBIC6* (*Gobar.A04 G079100*), *LjBIC1* (*Lj1g0014395*), *LjBIC2* (*Lj3g0006810*), and *LjBIC3* (*Lj5g0015488*).

### Vectors Construction

For the construction of overexpression vectors, the CDS sequences amplified from Ws82 cDNA were first cloned into the *pDONR-Zeo* vector and then cloned into the *pEarleyGate101* or *pEarleyGate104* vector using the Gateway recombinant system by BP reaction and LR reaction, respectively (Invitrogen) (Earley et al., 2006). For the construction of CRISPR/Cas9 vectors, at least three gRNA target sites were selected for each gene using the website tool CRISPRdirect^2^ (Naito et al., 2015). The soybean hair root system was used to test the efficiency of each gRNA, and then the effective gRNA was selected to construct the single or double knockout vectors (Li et al., 2020; Lyu et al., 2021). For the construction of subcellular localization vectors, the CDS of the indicted gene was cloned into the *pA7-YFP* or *pA7-RFP* vector using the In-fusion system (Clontech). The CDS of *GmMYB29* was cloned into the *pA7-RFP* vector as a nuclear marker (Chu et al., 2017). The *pA7-YFP* or *pA7-RFP* empty vector was used as a control. To construct vectors for yeast two-hybrid experiment, the CDS of the indicated gene was cloned into the *pGADT7* or *pBridge* vector using the In-fusion system, respectively.

### Soybean Transformation

The overexpression lines and CRISPR/Cas9-engineered mutants were obtained by the Agrobacterium-mediated soybean cotyledon nodule transformation method (Zhang et al., 1999). The general process is as follows: healthy seeds were selected and sterilized by chlorine for 16 h, then soaked into sterilized water overnight. The seed coat was gently removed, and the seeds were divided into two cotyledon explants. After being gently scratched at the cotyledon node, the explants were immersed in *Agrobacterium* (EHA105) which harbors expression vectors for 30 min and then transferred to the co-culture medium. After 3 days of co-culture, the explants were washed by sterilized water with antibiotics to remove the bacteria on the surface, transferred to the shoot initiation medium and subcultured once for 10 days with three repetitions. The explants with tufted shoots were then transferred to shoot elongation medium and subcultured once for 10 days with three repetitions. The elongated shoots were cut and moved to the rooting medium. The shoot initiation medium and shoot elongation medium contain glufosinate (5 mg/L) to screen positive transgenic shoots.

### Subcellular Localization in Soybean Mesophyll Protoplasts

To investigate the subcellular localization of GmBICs protein, the *GmBICs-YFP* or *pA7-YFP* empty vectors were transferred into soybean protoplasts together with *GmMYB29-RFP* (nuclear marker) according to the previously described method (Chu et al., 2017; Xiong et al., 2019). To explore whether GmBICs could inhibit GmCRYs photobodies, the *GmBIC-RFP* or *pA7-RFP* vector and *GmCRY-YFP* were co-transferred into soybean mesophyll protoplasts and cultured overnight in dark conditions, then exposed to blue light (25 μmol m^–2^s^–1^) for 5 min before fluorescence irradiation. Fluorescent images were captured by Zeiss LSM700 confocal laser scanning microscope, and the fluorescence of chloroplast, RFP, and YFP was excited by 639, 555, and 488 nm laser, respectively.

### Light Regimes

For the LBL treatment, white light (WL) was filtered through two layers of yellow filter film (no. 101, Lee Filters, CA, United States) as described previously (Lyu et al., 2021). The photosynthetically active radiation (400–700 nm) of both WL and LBL was set to 500 mmol m^–2^ s^–1^ as measured by HiPoint HR-350 Spectrometer.

### RNA Extraction and qRT-PCR

Total RNA was extracted with TRIzol reagent from quick-frozen and grounded soybean leaves. Then 4 mg of total RNA was used for reverse transcription of first-strand cDNAs by kit (TransScript II One-Step gDNA Removal and cDNA Synthesis SuperMix, TransGen) with the Oligo (dT)18 primer in 20 μl volume system. For qRT-PCR, 1.5 μl of 10 times diluted cDNA was used as the template for amplification using TB Green Premix Ex Taq (Takara) on Roche LightCycler 480 equipment following the manufacturer’s instructions. Three biological replicates were performed for each sample.

### Yeast Two-Hybrid Assay

For the yeast two-hybrid assay, the prey vector *pGADT7* expressing GmBIC1a and the bait vector *pBridge* expressing GmCRYs were co-transformed to the yeast strain AH109. The positive clones screened on the SD-LW plate were then incubated on the SD-LWHA plate in the dark or blue light (50 μmol m^–2^ s^–1^) conditions for 3 days at the temperature of 30°C.

## Results

### The Blue-Light Inhibitors of Cryptochrome Gene Family in Soybean

**FIGURE 1:**
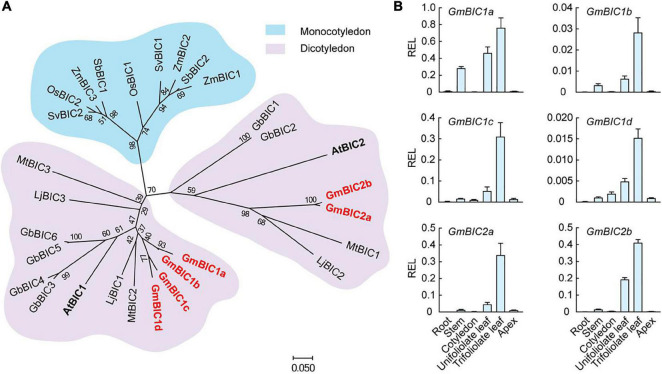
Phylogeny and tissue-specific transcriptional analysis of *GmBICs*. **(A)** Phylogenetic tree of GmBIC proteins with other plant BIC proteins using the neighbor-joining method by the MEGA7. The bootstrap analysis employed 1,000 replicates. Nomenclatures are as follows: At, *Arabidopsis thaliana*; Gm, *Glycine max*; Lj, *Lotus japonicus*; Mt, *Medicago truncatula*; Gb, *Gossypium barbadense*; Zm, *Zea mays*; Os, *Oryza sativa*; Sv, *Setaria viridis*; and Sb, *Sorghum bicolor*. **(B)** Relative expression levels of *GmBICs* in different tissues of Williams 82 by qRT–PCR. Data are means ± SD (*n* = 3). *GmActin* was used as an internal control. REL, relative expression level.

In contrast to cryptochrome present in organisms ranging from bacteria to humans, BIC is only found in land plants (Wang et al., 2017). We selected the *BIC* gene family in soybean, *Arabidopsis*, and other plants to construct the phylogenetic tree using the neighbor-joining method by the MEGA7 based on their protein sequences (Figure 1A). The BIC proteins were divided into the monocotyledon clade (shaded with blue) and the dicotyledon clade (shaded with pink). The dicotyledon BICs were further grouped into two subclades, containing AtBIC1 and AtBIC2, respectively. We total identified six BIC-like proteins in soybean. The four co-orthologs of *Arabidopsis* BIC1 were named GmBIC1a, GmBIC1b, GmBIC1c, and GmBIC1d, and the two co-orthologs of *Arabidopsis* BIC2 were named GmBIC2a and GmBIC2b, respectively. The alignments of the protein sequences indicate that all the soybean and *Arabidopsis* BIC proteins contain the conserved Cryptochrome Interacting Domain (CID) (Wang Q. et al., 2016; Ma et al., 2020), implying that the soybean GmBICs may function similarly as the *Arabidopsis* BICs in the cryptochrome mediated blue light signaling pathway (Supplementary Figure 1). Consistent with this speculation, GmBIC1a was able to physically interact with GmCRY1b and GmCRY2a in yeast and inhibit the GmCRY1a and GmCRY2a photobodies in soybean protoplasts (Supplementary Figure 2).

To investigate the expressional pattern of *GmBICs* in soybean, we performed a qRT-PCR assay using various tissues of Ws82 seedlings grown under continuous light. Overall, the six *GmBIC* genes showed similar expression patterns with the highest expression levels in the first trifoliolate leaf, followed by unifoliolate leaf, stem, cotyledon, and apical tissue. The expressions of *GmBICs* were extremely low in the root, implying that the GmBICs majorly function in the upground tissues that can access the light (Figure 1B).

### Subcellular Localization of GmBICs

**FIGURE 2:**
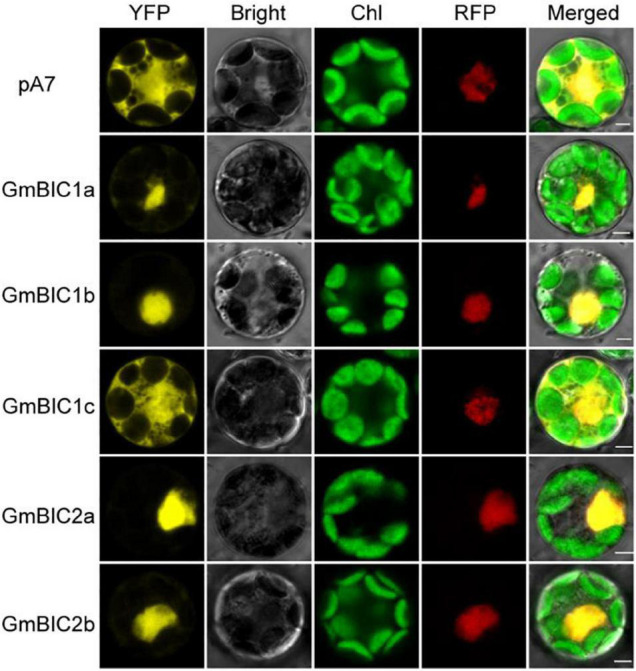
Subcellular localization of recombinant GmBIC proteins in soybean mesophyll protoplasts. Fluorescence image of subcellular localization of GmBICs-YFP proteins in mesophyll protoplasts prepared from the leaves of 12-day-old Williams 82 grown under short-day conditions. The GmMYB29-RFP fusion protein was used as the nuclear marker. *pA7-YFP* empty vector was used as a control. Scale bars = 2 μm.

To explore the subcellular localization of GmBICs, the recombinant vector encoding the GmBIC-YFP fusion protein under the control of the *35S CaMV* promoter was co-transferred into soybean mesophyll protoplasts with the vector expressing the GmMYB29-RFP nuclear maker. Confocal microscopy showed that the YFP fluorescence expressed by the control *pA7-YFP* vector was dispersed throughout the entire cells while the GmBIC1a-YFP, GmBIC1b-YFP, GmBIC2a-YFP, and GmBIC2b-YFP fusion proteins were mainly localized in the nuclear of the soybean cells, indicating that the GmBIC1a, GmBIC1b, GmBIC2a, and GmBIC2b are nuclear-localized proteins. In contrast, GmBIC1c-YFP was located in both the cytosol and the nucleus, implying that GmBIC1c may have evolved functions different to other GmBICs in soybean (Figure 2). The subcellular localization of GmBIC1d was not determined here because no fluorescent signal could be detected for the *GmBIC1d-YFP* construct under our experimental conditions.

### Overexpressions of GmBICs Promote Internode Elongation in Soybean

**FIGURE 3:**
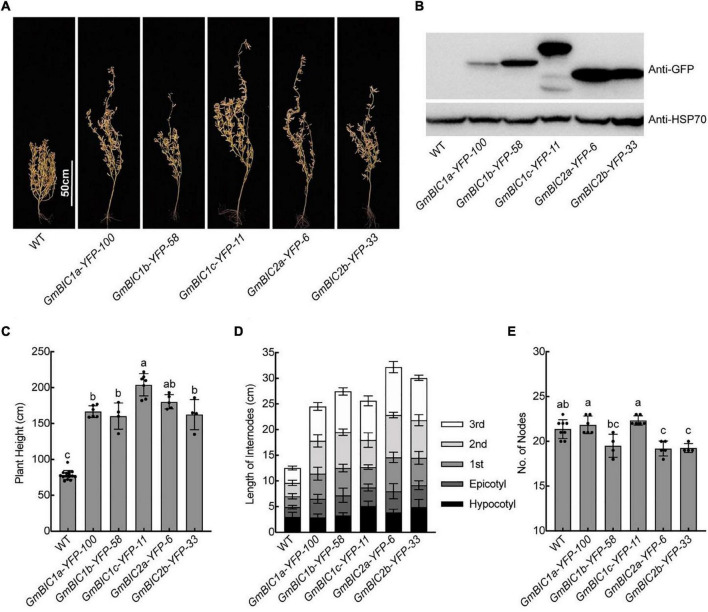
Plant height phenotypes of the *GmBICs-OX* lines in field. **(A)** Representative images of indicated lines at the maturation stage grown in the Beijing field in 2020. Homozygous overexpression lines at T4 or higher generation were used. Scale bars = 50 cm. **(B)** Immunoblots of the GmBIC-YFP fusion proteins of each line as in **(A)** probed with the anti-GFP antibodies. HSP70 proteins were used as the loading control. Statistical analysis of the plant height **(C)**, the internodes length **(D)**, and the nodes number **(E)** of each line as in **(A)**. Data are means ± SD (*n* ≥ 4). Lowercase letters indicate significant differences (*p* < 0.01, One-way ANOVA with Tukey’s multiple comparisons test).

To investigate the functions of GmBICs, we constructed the *35S:GmBICs-YFP* or *35S:YFP-GmBICs* vectors for stable genetic transformation of soybean cultivar TL1. We obtained at least two overexpression lines for *GmBIC1a*, *GmBIC1b*, *GmBIC1c*, *GmBIC2a*, and *GmBIC2b*, respectively. These overexpression lines confirmed by immunoblot assay were grown in the Beijing field and greenhouse for the phenotypic analysis (Figures 3A,B, Supplementary Figures 3A,B). The results showed that the plant heights of all lines were significantly increased (Figures 3A,C and Supplementary Figures 3A,C). We measured the length of hypocotyl, epicotyl, and the first to the third internodes. The results demonstrated that each internode significantly elongated in these overexpression lines in comparison to the wild type (WT) (Figure 3D). The nodes number was not increased by overexpressions of GmBICs or even decreased in some transgenic plants, including the *GmBIC1b-YFP-58, GmBIC2a-YFP-6*, and *GmBIC2b-YFP-33* lines (Figure 3E), suggesting that GmBICs action as plant height enhancer by promoting internode elongation rather than increasing node number in soybean. We also obtained multiple *35S:GmBIC1d-YFP* transgenic lines, but none of them displayed an obvious elongated phenotype. In consistent with this, the expression of GmBIC1d-YFP was detected neither in transiently transformed mesophyll protoplasts nor in stable transgenic lines.

### GmBICs Corporately Regulate Plant Height in Soybean

**FIGURE 4:**
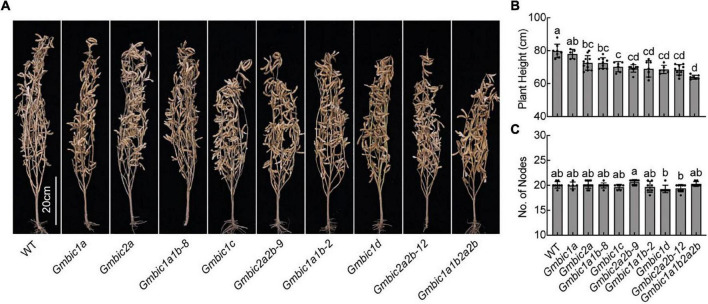
Plant height phenotypes of *Gmbic* mutants in field. **(A)** Representative images of indicated lines at the maturation stage grown in the Beijing field in 2021. Homozygous CRISPR/Cas9 knockout lines at T4 or higher generation were used. Scale bars = 20 cm. Statistical analysis of the plant height **(B)** and the number of nodes **(C)** of each line in **(A)**. Data are means ± SD (*n* > 5). Lowercase letters indicate significant differences (*p* < 0.05, one-way ANOVA with Tukey’s multiple comparisons test).

Next, we investigated the effect of GmBICs on plant height using the CRISPR/Cas9-engineered technology. The candidate gRNA targeting each *GmBIC* gene was designed by the website tool CRISPRdirect (see text footnote 2) (Naito et al., 2015) and then constructed into the CRISPR/Cas9 expression vector. The editing efficiency of each candidate gRNA was tested by the hairy root transformation system (Sun et al., 2015), and those constructs with high editing efficiency were selected for stable transformation (Supplementary Figure 4). A series of homozygous single and double mutants were generated, and the *Gmbic1a1b2a2b* quadruple mutant was further obtained by genetic crossing between the *Gmbic1a1b-2* and *Gmbic2a2b-12* double mutants (Figure 4A and Supplementary Figure 4).

We investigated the plant height trait at the maturation stage in the Beijing field. The result demonstrated that the above set of mutants displayed a progressive reduction in plant height trait with an order as shown in Figure 4A. Except for the *Gmbic1a* mutant, all other mutant lines were significantly dwarfing compared to the wild type (Figures 4A,B). The *Gmbic1a1b-2* and *Gmbic1a1b-8* double mutants were shorter than the *Gmbic1a* single mutant, suggesting that the *GmBIC1a* and *GmBIC1b* genes are functionally redundant in promoting stem elongation. The observation that the *Gmbic1a1b-8* mutant was shorter than the *Gmbic1a* mutant but taller than the *Gmbic1a1b-2* mutant is possibly due to the fact that the *GmBIC1a* gene in the *Gmbic1a1b-8* mutant harbors a 3 bp deletion in the coding DNA sequence (CDS) without frameshift (Supplementary Figure 4). Notably, the *Gmbic1c* and *Gmbic1d* mutants were almost as dwarf as the *Gmbic1a1b* and *Gmbic2a2b* mutants, suggesting that the *GmBIC1c* and *GmBIC1d* genes are more prominent in regulating plant height than other *GmBIC* genes (Figures 4A,B). The *Gmbic1a1b2a2b* quadruple mutant showed a further reduced plant height phenotype, suggesting the redundant/additive roles of GmBICs in regulating stem elongation. The node numbers of all mutants displayed no apparent differences compared to WT (Figure 4C), confirming that the *GmBIC* genes regulate plant height by promoting internode elongation rather than modulating node numbers in soybean.

### GmBICs Involved in Low Blue Light-Induced Stem Elongation in Soybean

**FIGURE 5:**
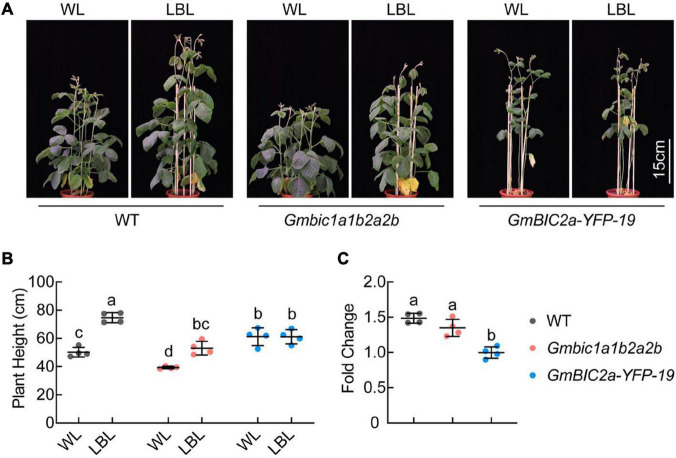
GmBICs involved in LBL-induced stem elongation in soybean. **(A)** Representative images of the indicated lines grown under WL and LBL conditions. De-etiolated 10-day-old seedlings were treated with the indicated light regimes for 14 days under long-day conditions. Scale bar = 15 cm. **(B)** Statistical analysis of the plant height of each line in **(A)**. Data are means ± SD (*n* = 4). The lowercase letters indicate significant differences (*p* < 0.05, two-way ANOVA with Tukey’s multiple comparisons test). **(C)** Statistical analysis of fold change in plant height of each line grown under LBL relative to that under WL, as in **(A)**. Data are the means ± SD (*n* = 4). The lowercase letters indicate significant differences (*p* < 0.01, one-way ANOVA with Tukey’s multiple comparisons test). WL, white light; LBL, low blue light.

Our previous study showed that GmCRY1s mediate LBL-induced stem elongation in soybean (Lyu et al., 2021). The *GmBICs-OX* lines and the CRISPR/Cas9 knockout mutants are phenotypically reminiscent to the *Gmcry1s* mutants and the *GmCRY1s-OX* lines, respectively, suggesting that GmBICs promote the LBL-induced stem elongation by antagonizing GmCRY1s. To assess the role of GmBICs in this process, we compared the *GmBICs-OX* lines and the *Gmbic* mutants with the WT plants in response to LBL treatment. The de-etiolated 10-day-old seedlings were treated with WL or LBL (blue light was removed by two layers light filter) under long-day conditions (16 h light/8 h dark) for 14 days. The *GmBIC2a-OX* line failed to respond to LBL and showed the same plant height under both WL and LBL conditions. In contrast, the WT and *Gmbic1a1b2a2b* quadruple mutant showed significantly increased plant height under LBL, and the plant height of *Gmbic1a1b2a2b* was shorter than that of WT under both WL and LBL conditions (Figures 5A,B). Moreover, the fold change (the plant height of each line grown under LBL relative to that under WL) of the *Gmbic1a1b2a2b* was lower than that of WT (Figure 5C). The status of the *Gmbic1a1b2a2b* quadruple mutant under LBL is reminiscent to that of the *GmCRY1b-OX* lines, implying a potential of utilizing the *Gmbic* mutants to breed shade-tolerant soybean cultivars.

## Discussion

Soybean is the most important legume crop, providing 61% of oilseed production and 70% of protein meal across the world (Lee et al., 2019; SoyStat, 2019). The total yield of soybean needs to increase 2.4% per year to catch the demands of the ever-growing world population (Ray et al., 2012, 2013). Intercropping and high-density planting have been widely used to increase soybean production. However, these two cultivation modes usually induce unfavorite SAS (Libenson et al., 2002; Egli and Bruening, 2005; Ballare et al., 2012; Wang H. et al., 2016), especially the exaggerated stem elongation that confers serious lodging and reduction of yield over 20% in soybean (Noor and Caviness, 1980; Cober et al., 2005; Carriedo et al., 2016; Liu et al., 2017).

Recently, we reported that GmCRY1s mediate the LBL signal to regulate stem elongation in soybean. Overexpression of GmCRY1s reduced the extent of stem elongation and significantly elevated soybean yield under high-density conditions, which demonstrated a practicable way to breed lodging-resistant and high yield soybean cultivars by enhancing the blue light signaling transduction activities (Lyu et al., 2021). Here, we further test this possibility by investigating the functions of *GmBIC* genes which are supposed to be cryptochrome antagonists in soybean.

We found that although the six *GmBIC* genes have similar expression profiles in various tissues (Figure 1B), the GmBIC proteins showed different subcellular localization patterns: the GmBIC1a, GmBIC1b, GmBIC2a and GmBIC2b proteins were only detected in the nucleus, while the GmBIC1c protein was distributed in both cytosol and nucleus as the *Arabidopsis* BIC1 and BIC2 proteins (Wang et al., 2017). Moreover, knockout of individual *GmBIC* gene conferred different extent of dwarfing phenotypes with the most obvious *Gmbic1d* mutant, followed by the *Gmbic1c*, *Gmbic2a* and *Gmbic1a* mutants. These observations suggested that the six *GmBIC* genes may have evolved special functions in regulating soybean growth and development.

We further showed that the single, double and quadruple *Gmbic* mutants displayed progressively severe dwarf phenotypes, by decreasing the internode length rather than reducing node number. It could be speculated that the *Gmbic* pentadruple and hexatruple mutants will further reduce the plant height. The *Gmbic* quadruple mutant maintained the advantage of dwarfing under LBL conditions where the stem of the WT soybean was extremely elongated. These results suggested that under high-density planting conditions, the *Gmbic* mutant can effectively alleviate the stem elongation caused by shading of surrounding plants, so as to avoid lodging. Further studies are necessary to elucidate the mechanisms of how GmBICs regulate stem elongation, and to assess the potential of precisely designing plant height through modulating *GmBIC* genes in soybean.

## Data Availability Statement

The original contributions presented in the study are included in the article/Supplementary Material, further inquiries can be directed to the corresponding author/s.

## Author Contributions

BL designed the research. RM, XL, RJ, JL, TZ, and HL performed the experiments. RM and XL collected the phenotypic data. RM analyzed the data. BL and RM wrote the manuscript. All authors contributed to the article and approved the submitted version.

## Conflict of Interest

The authors declare that the research was conducted in the absence of any commercial or financial relationships that could be construed as a potential conflict of interest.

## Publisher’s Note

All claims expressed in this article are solely those of the authors and do not necessarily represent those of their affiliated organizations, or those of the publisher, the editors and the reviewers. Any product that may be evaluated in this article, or claim that may be made by its manufacturer, is not guaranteed or endorsed by the publisher.

## Footnotes

1
https://phytozome-next.jgi.doe.gov

2
http://crispr.dbcls.jp/
