# Supplementary material for: GmBICs Modulate Low Blue Light-Induced Stem Elongation in Soybean
Source: Front Plant Sci. 2022 Feb 3;13:803122. doi: 10.3389/fpls.2022.803122 (PMC8850649; doi:10.3389/fpls.2022.803122)
Supplement: Supplementary file 2 [file Image_1.pdf]

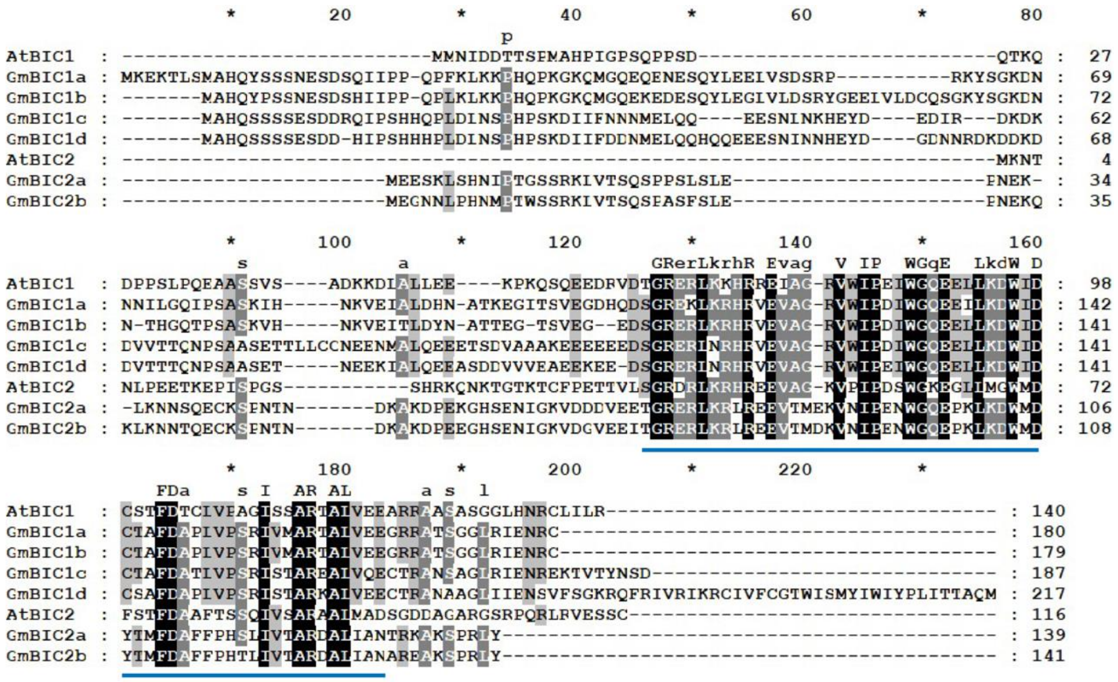

SUPPLEMENTARY FIGURE S1 | Sequence alignment of BIC proteins in *Arabidopsis* and soybean. The letter with a blue underline indicates the CRY-Interacting Domains (CID).

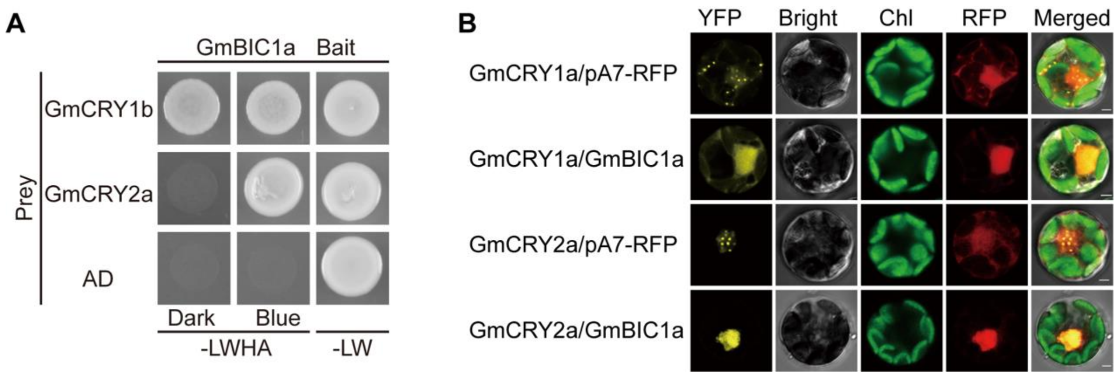

SUPPLEMENTARY FIGURE S2 | GmBIC1a interacts with GmCRYs and inhibits the formation of GmCRY photobodies. **(A)** Interaction of GmBIC1a with GmCRY1b and GmCRY2a in yeast. **(B)** GmBIC1a inhibition of GmCRY1a-YFP and GmCRY2a-YFP photobodies in soybean mesophyll protoplasts. The protoplasts were exposed to blue light (25 μmol $m^{-2}s^{-1}$) for 5 min and examined under a fluorescence microscope. The *pA7-RFP* empty vector was used as a control. Scale bars = 2 μm.

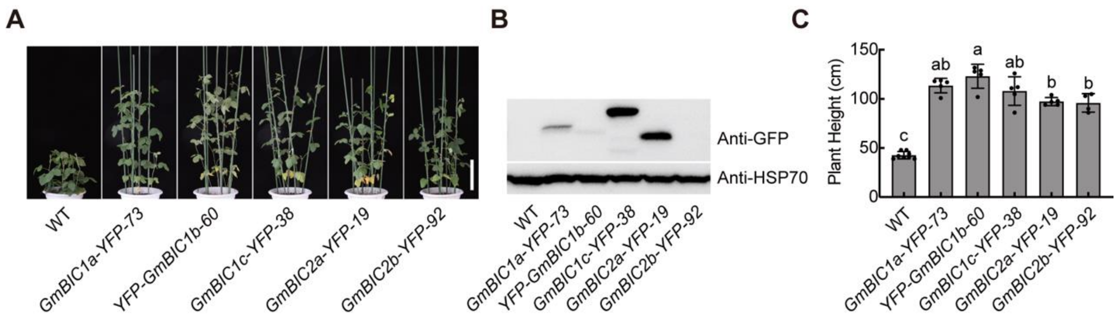

SUPPLEMENTARY FIGURE S3 | Phenotypes of the *GmBIC* overexpression lines. **(A)** Representative images of indicated lines grown under short-day conditions (8 h light/16 h dark). Scale bars = 25 cm. **(B)** Immunoblots of the GmBIC-YFP or YFP-GmBIC fusion proteins of each line as in **(A)** probed with the anti-GFP antibodies. HSP70 proteins were used as the loading control. **(C)** Statistical analysis of the plant height of each line as in **(A)**. Data are means ± SD ($n \geq 4$). Lowercase letters indicate significant differences ($p < 0.01$, One-way ANOVA with Tukey's multiple comparisons test).

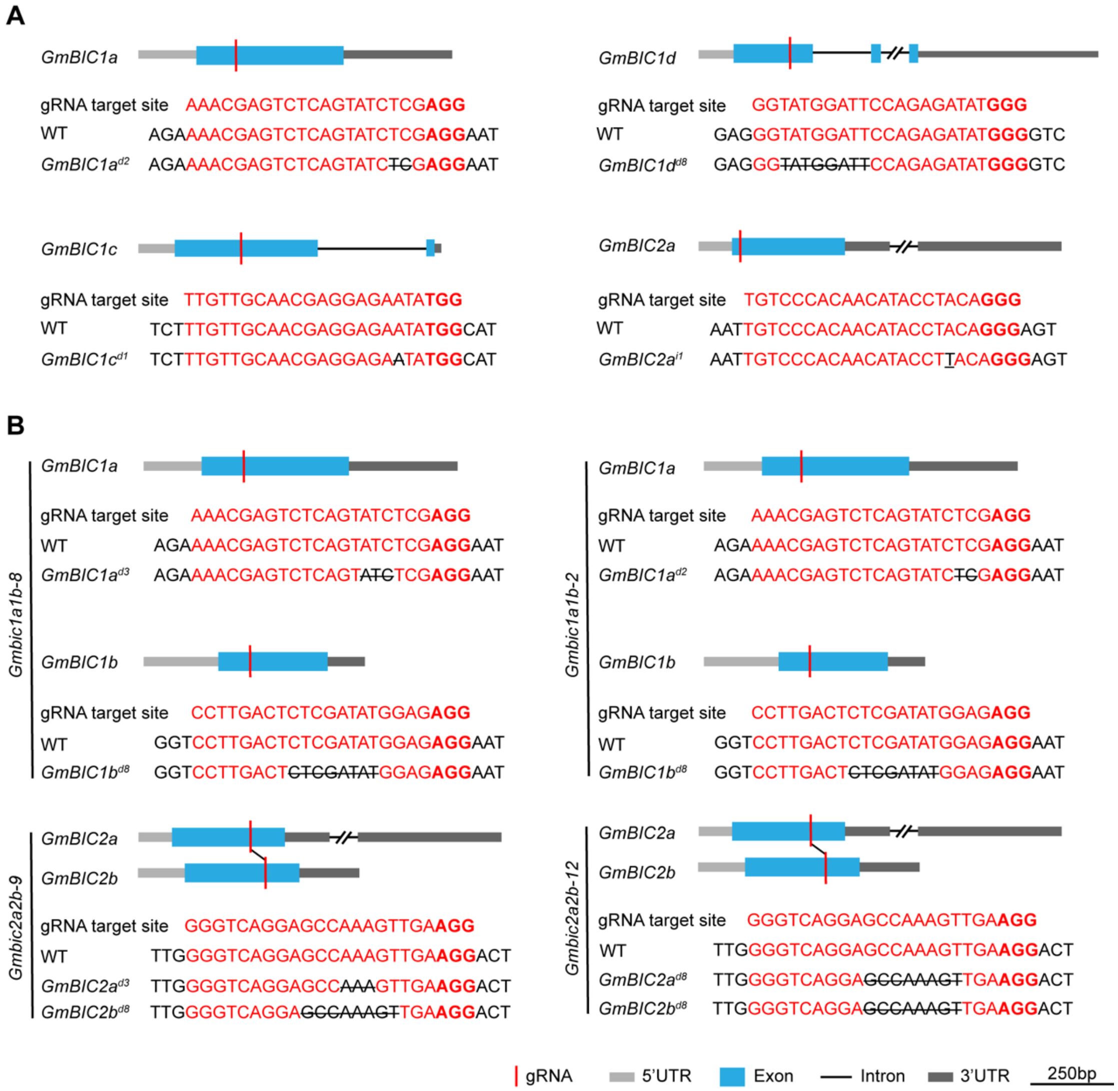

SUPPLEMENTARY FIGURE S4 | Schematic diagram of the *Gmbic* mutations generated by CRISPR/Cas9. **(A and B)** Schematic diagram showing the genomic structures, the gRNA-targeting sites and the detailed editing sequence of *GmBIC* genes in the *Gmbic1a*, *Gmbic1c*, *Gmbic1d*, and *Gmbic2a* single mutants **(A)**, and the *Gmbic1a1b* and *Gmbic2a2b* double mutants **(B)**. The underlined and strikethrough letters indicated the inserted and deleted nucleotides, respectively. The gRNA-targeting sites are indicated in red.
